# Supplementary material for: Synthesis, Structure, and Magnetic and Biological Properties of Copper(II) Complexes with 1,3,4-Thiadiazole Derivatives
Source: Int J Mol Sci. 2023 Aug 21;24(16):13024. doi: 10.3390/ijms241613024 (PMC10455747; doi:10.3390/ijms241613024)
Supplement: Supplementary file 1 [file ijms-24-13024-s001.zip › ijms-2487706-supplementary.pdf]

Supplementary Information

Synthesis, structure, and magnetic and biological properties of copper(II) complexes with 1,3,4-thiadiazole derivatives

Lyudmila G. Lavrenova, Taisiya S. Sukhikh, Lyudmila A. Glinskaya, Svetlana V. Trubina, Valentina V. Zvereva, Alexander N. Lavrov, Lyubov S. Klyushova, Alexander V. Artem’ev

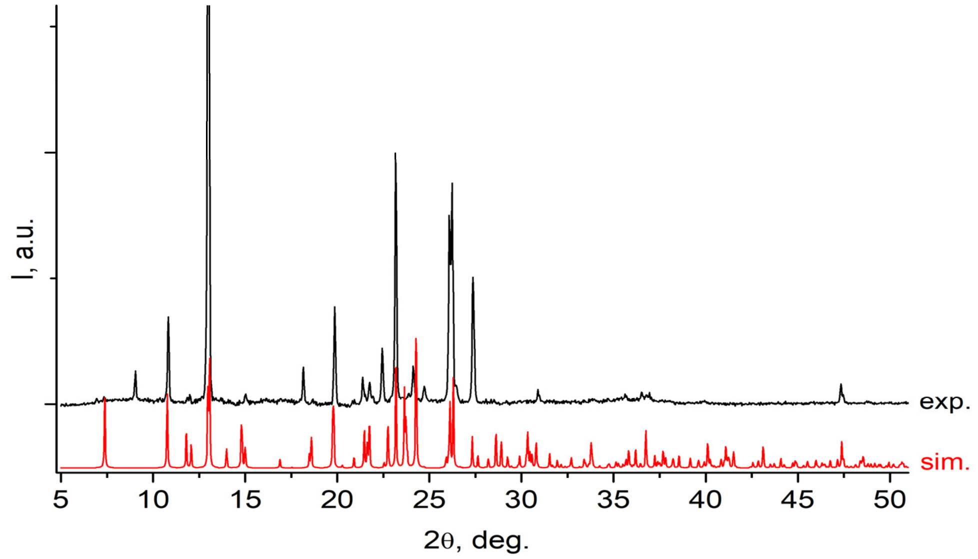

Figure S1. Experimental and simulated XRD powder patterns for $Cu(L^1)_2Br_2$.

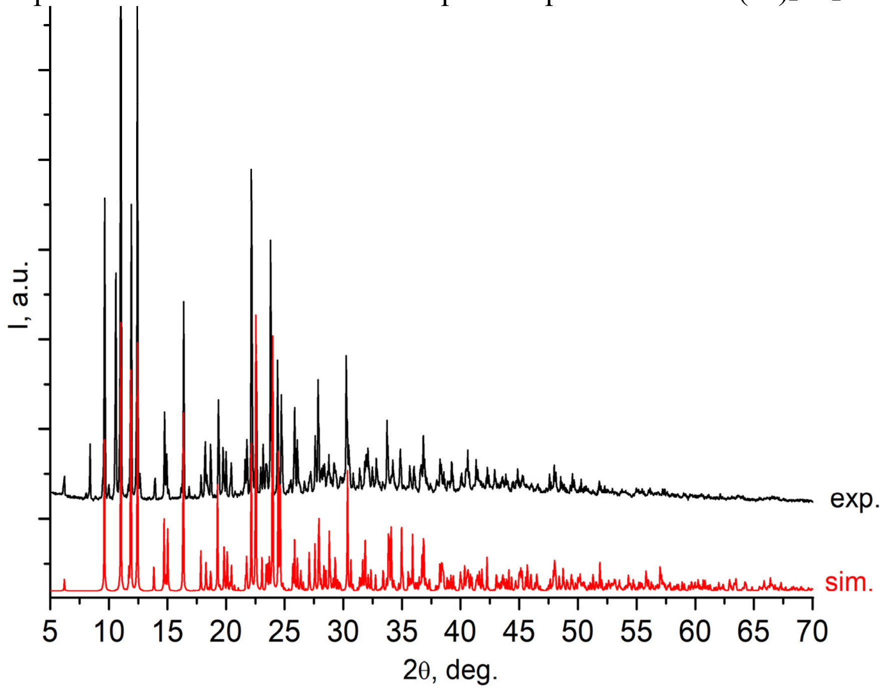

Figure S2. Experimental and simulated XRD powder patterns for $Cu(L^1)_2(C_2N_3)_2$.

Table S1. CSD 5.44 (April 2023) survey of crystal structures comprising a fragment {CuL_xBr_y}, where L = N heterocyclic ligand with the N having three neighbors (i.e. the N is sp^2-hybridized), x, y = 2–4. The coordination number (CN) is restricted to be equal of selected environment.

	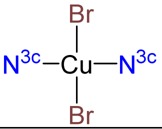	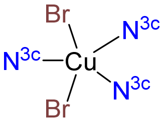	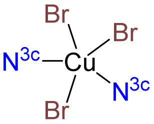	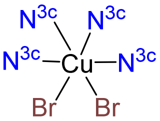	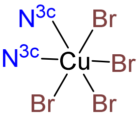	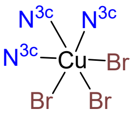
CN	4	5	5	6	6	6
hits	268	73	51	29	8	2

Table S2. CSD 5.44 (April 2023) survey of crystal structures comprising a fragment {$CuN_x(C_2N_3)$}, where x = 3–5, with any κ-N-ligands. The coordination number (CN) is restricted to be equal of selected environment.

	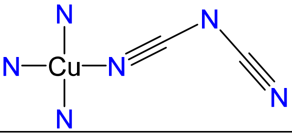	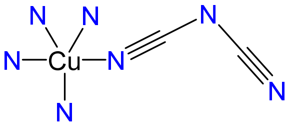	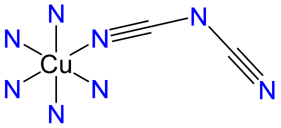
CN	4	5	6
hits	10	61	24

Table S3. Crystal data and structure refinement details for **1** and **2**

Parameter	**1**	**2**
Empirical formula	$C_{12}H_{20}Br_2CuN_4S_6$	$C_{10}H_{10}N_8S_3Cu$
Molecular weight	636.04	401.99
Crystal system	Triclinic	Triclinic
Space group	*P*-1	*P*-1
a, Å<br>*b*, Å<br>*c*, Å<br>α,°<br>β,°<br>γ,°	8.6690(3)<br>9.8723(3)<br>14.6229(4)<br>80.407(1)<br>75.956(1)<br>67.320(1)	7.8405(6)<br>8.6679(4)<br>12.1902(9)<br>97.763(2)<br>95.598(2)<br>108.517(2)
V, $Å^3$	1116.44(6)	769.62(9)
Z; D_{calc}, g/cm^3	2, 1.892	2, 1.745
μ, mm^{-1}	5.124	1.833
Crystal dimensions, mm	0.15x0.05x 0.02	0.14x0.14x 0.08
2θ Range for data collection, deg.	5.13-63.14	5.04-61.04
Reflections collected / unique	27939<br>7452	15207<br>4621
Reflections with $I > 2\sigma(I)$	6563	4147
Refined parameters	244	201
GOOF on F^2	1.052	1.035
Final *R* indices [$I > 2\sigma(I)$] R_1, wR_2	0.0252<br>0.0515	0.0307<br>0.0670
R indices (all data) R_1, wR_2	0.0314<br>0.0536	0.0352<br>0.0694
Largest diff. peak and hole, e/$Å^3$	1.19/ –1.01	0.53/ –0.50

Table S4. Main interatomic distances and bond angles in the structures of **1** and **2**

**1**		**2**	
Bond	d,Å	Bond	d,Å
Cu1–Br1	2.3725(3)	Cu1–N2	2.2427(15)
Cu1–Br2	2.3800(3)	Cu1–N5[1]	1.9744(16)
Cu1–N2	1.9739(13)	Cu1–N3	1.9814(15)
Cu1–N2A	1.9837(14)	Cu1–N5B	1.9740(16)
S2–C3	1.728(2)	Cu1–N3B[2]	1.9789(15)
S2–C4	1.739(2)	S2–C4	1.7295(17)
S2A–C3A	1.723(2)	S2–C3	1.7406(18)
S2A–C4A	1.737(2)	S3–C4	1.7431(17)
S1–C3	1.731(2)	S3–C5	1.8133(19)
S1–C2	1.819(2)	S1–C2	1.812(2)
S1A–C3A	1.731(2)	S1–C3	1.738(2)
S1A–C2A	1.820(2)	N2–N1	1.390(2)
S3–C4	1.734(2)	N2–C4	1.304(2)
S3–C5	1.814(2)		
Angle	ω, deg.	Angle	ω, deg.
Br1–Cu1–Br2	152.34(1)	N5[1]–Cu1–N2	96.73(6)
N2–Cu1–Br1	91.98(4)	N3–Cu1–N2	98.65(6)
N2–Cu1–Br2	92.15(4)	N3–Cu1–N5[1]	88.32(6)
N2–Cu1–N2A	158.30(6)	N5B–Cu1–N2	94.27(6)
N2A–Cu1–Br1	93.30(4)	N5B–Cu1–N5[1]	169.00(7)
N2A–Cu1–Br2	92.87(4)	N5B–Cu1–N3	90.56(6)
C3–S2–C4	87.17(8)	N3B[2]–Cu1–N2	95.06(6)
C3A–S2A–C4A	87.24(8)	N3B[2]–Cu1–N5[1]	91.00(6)
C3–S1–C2	101.72(8)	N3B[2]–Cu1–N3	166.25(7)
C3A–S1A–C2A	102.11(9)	N3B[2]–Cu1–N5B	87.50(6)

[#1] 1-X, 1-Y, 2-Z; [#2] –X, -Y, 1-Z
